# Cardiolipin, Perhydroxyl Radicals, and Lipid Peroxidation in Mitochondrial Dysfunctions and Aging

**DOI:** 10.1155/2020/1323028

**Published:** 2020-09-08

**Authors:** Alexander V. Panov, Sergey I. Dikalov

**Affiliations:** ^1^Federal Scientific Center for Family Health and Human Reproduction Problems, 16 Timiryasev str., Irkutsk, 664003, Russian Federation, Russia; ^2^Division of Clinical Pharmacology, Vanderbilt University Medical Center, Nashville, TN 37232, USA

## Abstract

Mitochondrial dysfunctions caused by oxidative stress are currently regarded as the main cause of aging. Accumulation of mutations and deletions of mtDNA is a hallmark of aging. So far, however, there is no evidence that most studied oxygen radicals are directly responsible for mutations of mtDNA. Oxidative damages to cardiolipin (CL) and phosphatidylethanolamine (PEA) are also hallmarks of oxidative stress, but the mechanisms of their damage remain obscure. CL is the only phospholipid present almost exclusively in the inner mitochondrial membrane (IMM) where it is responsible, together with PEA, for the maintenance of the superstructures of oxidative phosphorylation enzymes. CL has negative charges at the headgroups and due to specific localization at the negative curves of the IMM, it creates areas with the strong negative charge where local pH may be several units lower than in the surrounding bulk phases. At these sites with the higher acidity, the chance of protonation of the superoxide radical (O_2_^•^), generated by the respiratory chain, is much higher with the formation of the highly reactive hydrophobic perhydroxyl radical (HO_2_^•^). HO_2_^•^ specifically reacts with the double bonds of polyunsaturated fatty acids (PUFA) initiating the isoprostane pathway of lipid peroxidation. Because HO_2_^•^ is formed close to CL aggregates and PEA, it causes peroxidation of the linoleic acid in CL and also damages PEA. This causes disruption of the structural and functional integrity of the respirosomes and ATP synthase. We provide evidence that in elderly individuals with metabolic syndrome (MetS), fatty acids become the major substrates for production of ATP and this may increase several-fold generation of O_2_^•^ and thus HO_2_^•^. We conclude that MetS accelerates aging and the mitochondrial dysfunctions are caused by the HO_2_^•^-induced direct oxidation of CL and the isoprostane pathway of lipid peroxidation (IPLP). The toxic products of IPLP damage not only PEA, but also mtDNA and OXPHOS proteins. This results in gradual disruption of the structural and functional integrity of mitochondria and cells.

## 1. Introduction

For the last five decades, the free radical theory of aging, first proposed by Harman [1–3], was regarded as one of the most important and investigated among other hypotheses of aging. Accumulation of mtDNA point mutations [4] and an exponential increase with age of mtDNA deletions [5] were considered as important hallmarks of ageing and the age-related diseases. Accumulation of mtDNA mutations with increasing age was demonstrated mainly in heart, skeletal muscles, and brain, which undergo a high range of workloads and associated metabolic activities. In the liver, which has relatively stable high rate of metabolism and high regeneration capacity, there was shown no significant accumulation of mtDNA mutations with age [6]. For decades, it was generally agreed that ROS-associated somatic mutations of mtDNA contribute to human aging and to the decline of energetic capabilities at higher age [5, 7].

In recent years, however, new experimental data have discredited to some extent the original theory of direct participation of free radicals in mtDNA mutations [8–10]. It has also been found that most common ROS species either are not enough chemically active (O_2_^•^, ^•^NO) to initiate mutations or excessively active (^•^OH) and have a too short life span in order to reach mtDNA [11, 12]. Several decades ago, researchers from the Vanderbilt University have discovered the isoprostane pathway of lipid peroxidation (IPLP), which results in the formation of prostaglandin-like compounds with enormous variations in molecular positional isomerism and stereoisomerism [13–16]. Considering the chemistry of IPLP, we have proposed the mechanism of initiation of IPLP involving formation in the inner mitochondrial membrane of perhydroxyl radical (HO_2_^•^), which is the protonated form of superoxide radical (O_2_^•^) [12, 17]. We suggest that the mechanisms of action of perhydroxyl radical (HO_2_^•^) can explain the mechanism of initiation of the autoxidation of polyunsaturated fatty acids that are still part of the membrane's phospholipid. In addition, HO_2_^•^ can be regarded as a “carrier” of the highly toxic hydroxyl radical (^•^OH) inside the hydrophobic core of the membrane and thus can oxidize cardiolipin. This can happen in spite the fact that normally CL contains four linoleic acids with only two double bonds, which are not substrates for IPLP. In this review, we will focus on the roles of cardiolipin in oxidative stress due to the ability of this phospholipid to create, close to the surfaces of the inner mitochondrial membrane, the narrow layer of structured water with low pH and high conductance for protons [18–20]. This facilitates conversion of O_2_^•^, produced by the respiratory chain, into HO_2_^•^ that initiate IPLP [12, 17]. We consider that formation of toxic products, such as isolevuglandins (aka isoketals), produced by IPLP, as the major event in oxidative stress that is responsible for the slow but inevitable process of aging. The gradual accumulation of oxidative damages to mitochondrial enzymes and the membrane phospholipids, first of all phosphatidylethanolamine and cardiolipin, causes disruption of the structural and functional integrity of the respirosomes and ATP synthase.

## 2. Properties of Cardiolipin

Cardiolipin (CL) is the only phospholipid that is present almost exclusively in the inner mitochondrial membrane of all animals and in the plasma membrane of aerobic bacteria. That is in the membranes, where ATP is formed by the F_0_F_1_ ATP synthase [21]. CL is absent in the thylakoids of plants, where it is substituted by the plant sulfolipid (Sulfoquinovosyl dipalmitoylglycerol), probably because chloroplasts have another isoform of ATP-synthase (CF_0_CF_1_) [22], and, most importantly, the reversed, as compared to mitochondria, orientations of the energy-transforming enzymes, pH gradients, and electrical charges. The tight connection between the F_0_F_1_-ATP-syntahse and cardiolipin was proved by experiments, in which facultative anaerobes were placed into the oxygen-depleted atmosphere, and after a while, they lost not only F_0_F_1_-ATP-syntahse, but also cardiolipin [21].

**Figure 1:**
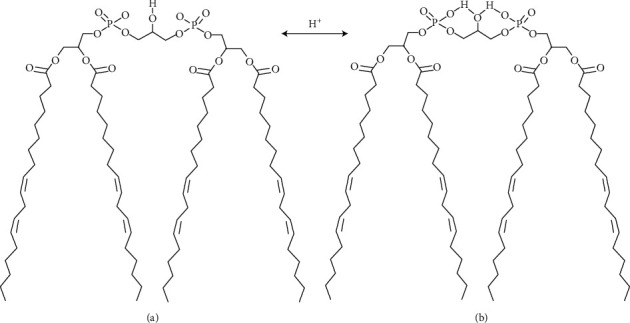
Cardiolipin structure. The figure depicts (for simplicity only) one of the cardiolipins–tetraoleilcardiolipin, and the two models headgroup ionization state at physiological pH 7.2. (a) Both phosphates have values of pK_a1_ of orthophosphoric acid. It is assumed that both phosphates ionize independently and the headgroup exists as a dianion. (b) Disparate pKa values of the two phosphates presume that the headgroup exists as a monoanion. The figure was adapted from [27]. Note: values for dissociation constants (pKa) of orthophosphoric acid: pK_a1_ = 2.15, *рК*_*а*2_ = 7.20, *ирК*_*а*3_ = 12.35.

The structure of the cardiolipin molecule was thoroughly described and discussed in [21, 23, 24]. The significance of CL specifically for the energy-transforming membranes comes from several intrinsic properties of the phospholipid, which originate from its unique structure (Figure 1). Due to the conical form, CL, together with also conical phosphatidylethanolamine (PEA), supports the superstructural organization of the respiratory chain and ATP synthase and allows accommodation of the multienzyme complexes into the sharp curves of the inner membrane. Due to the conical form and strong negative charges at the headgroups, CL has the propensity to form inverted hexagonal (HII) structures, which are essential for activation of the respiratory chain enzymes and ATP synthesis by promoting binding and conductance of protons through the hydrophobic milieu of the inner membrane [25–28] (Figure 1).

Figure 1 does not reflect fully the fact that the shape of CL resembles that of a pyramid or cone with the broad base and a very small head, which has the length of a glycerol molecule with three C atoms. In most cardiolipins, the fatty acids have usually two double bonds and therefore are strongly bend. For this reason, CL, similar to PEA, cannot form “normal” bilayer membranes [25, 27]. The two phosphate groups give the CL molecule the ability to trap protons [29] and thereby to facilitate proton translocation along the membrane surface [30].

In general, it has been found that composition of the four fatty acids in CL has small variations in the number of double bonds for the given organism or even tissue [31]. The selection of fatty acids in CL is specific for every species and organ, but unsaturated fatty acids are most common [32]. In many animals, the “mature” CL, that is after remodeling, has four linoleic acids with two double bonds (С18:2). In the fast respiring mitochondria of animals, CL may comprise up to 20% of all lipids, and up to 80% of CL is located in the inner leaflet of the inner mitochondrial membrane [33]. In the liver mitochondria, which have relatively low respiratory activity, CL is distributed more or less evenly between the two sides of the inner membrane. The unique structure of cardiolipin results in a number of important properties [23, 34] briefly listed as follows:

1. As the two small molecules of phosphatidic acid are located at the small “head” of the phospholipid, which is much smaller than the charged heads of other phospholipids, namely, phosphatidylcholine (PC), phosphatidylserine (PS), or phosphatidylglycerol (PG), the four fatty acids of CL are closer to each other with stronger attachments, and thus the temperature of phase transitions is higher than in the surrounding membrane. In other words, the lipids of CL are solid in comparison with the fluid lipids of surrounding phospholipids, most of which contain polyunsaturated fatty acids (PUFA). This allows protein complexes “float” being imbedded into the rafts, made of cardiolipin, and thus rearrange into functional supercomplexes, such as respirosomes
2. Small charged heads of CL allow the phospholipid to fit proteins into negative curvatures of the inner leaflet (the matrix side) of the inner membrane
3. Under physiological conditions, the presence of one or two negative charges at the phosphate groups allows CL to create the negatively charged regions that electrostatically interact with proteins and peptides. The excessive negative charges on the surface of rafts, comprised of many CL molecules, create the so-called antennae, when the Coulomb radii of several cardiolipins overlap and amplify a local negative charge
4. The mobility of the head group of CL is restricted because of binding of the glycerol to two phosphatidates. Therefore, the self-shielding of phosphate groups in CL is much lower than in other phospholipids in the membrane. As a result, the phosphates of cardiolipin on the surface of the inner membrane are easily accessible for interactions with proteins, peptides, and ions, as compared with other membrane phospholipids [23]. The interactions of CL with some proteins are so strong that CL was present in the crystal structures of the isolated mitochondrial proteins, for example, complex 3 and ANT [23]. Moreover, CL is known to specifically coordinate divalent cations with high affinity [23], and the ion:lipid stoichiometry is dependent on the CL headgroup formal charge [35].

It was suggested that distribution of the headgroup charge determines the types of nonbonding interactions that can occur among lipids, including electrostatic and hydrogen-bonding interactions [36, 37]. Clearly, for each of the above phenomena, the ionization state of CL headgroup is of fundamental importance; whether this lipid exists in the mono- or dianionic state, the charge strongly influences behavior of CL in the bilayer [27].

## 3. Formation of Cardiolipin Clusters with the Negatively Charged Antennae

The ability of CL to accommodate the sharp curvatures of the mitochondrial cristae explains the fact that most of CL molecules are specifically located in the inner leaflet of the inner mitochondrial membrane (IMM) and non-covalently linked to various proteins. The list of proteins linked to CL is very long and includes, first of all, the electron transport complexes of the respiratory chain, ATP-synthase, and a number of transmembrane carriers, such as ATP/ADP carrier (ANT), inorganic phosphate carrier, and uncoupling protein [26, 38]. A relatively small amount of CL, which is present in the outer leaflet of the inner mitochondrial membrane, was found to be bound to cytochrome *c*, and at the contact sites of the inner and outer membranes, CL interacts with creatine kinase and ANT [reviewed in 38].

From studies of the proteins crystals, it was deduced that CL construct bridges between proteins and thus promote existence of many proteins as dimers. It is known that all respiratory chain complexes and ATP-synthase are present in the membrane as homodimers, which, in their turn, are assembled into supercomplexes [39]. One of the consequences of the unique structure of CL is that it tends to form rafts by clustering at the sites with negative curvature of the IMM. At these sites, CL segregates laterally from other phospholipids and stabilizes the membrane, simultaneously with holding together the respirosomes [38, 40]. Although CL is the main anionic phospholipid comprising up to 20% of all phospholipids of the IMM [33], since it is located predominantly in the inner leaflet of the IMM, particularly at the negative curves of the cristae, at some locations, concentration of CL may be high. The regions with overlapping Coulomb radii of negative charges, called antennae, are rather common all over the matrix side of the IMM [33, 41].

The phosphoester groups of most phospholipids behave as strong acids with рК_а_ between 1 and 3, which coincide with the рК_а1_ of the orthophosphoric acid [27]. Therefore, in the absence of the intramolecular and intermolecular interactions, which could shield the phosphates at the head of CL, it could be expected that both phosphates of CL behave similarly, and that at the neutral pH of a cell CL behaves as a dianion [27]. Indeed, some researchers observed CL as a dianion with рК_а1_ ≈ 2 for both phosphates [27], whereas others observed large difference between рК_а1_ ≈ 2 and рК_а2_ ≈ 8 [21] and CL behavior as a monoanion. According to Haines [21], the reason for the discrepancies was caused by different properties of CL in the artificial membranes [27] and in the biological membranes [21].

Under normal conditions, the *Δ*pH between the cytosolic and the matrix sides of the inner mitochondrial membrane is close to 1.0, which is equivalent to 60 mV [42, 43]. It was suggested that one of the consequences of different dissociation of the CL phosphates may be as follows: on the cytosolic side (the outer surface of the IMM) CL traps protons, and thus converts *Δ*pH into Δ*Ψ*, by enhancing the “+” charge on the outer surface of the inner membrane. On the matrix side, CL behaves as a dianion, and thus enhances the “−” charge of the Δ*Ψ* at the inner leaflet of the IMM [28].

CL has an important function of buffering protons, particularly on the matrix side of the inner membrane [44]. In most active mitochondria of the heart, brain, skeletal muscles, and kidney, it is hardly possible to talk about pH of the matrix because mitochondrial proteins, together with water, exist as a quasicrystalline phase [45]. Antonenko et al. [46] suggested the existence of a kinetic barrier for proton transfer from the surface of bilayer phospholipid membrane to bulk water. Thus, there is no “normal” diffusion of protons and substrates in the bulk volume of the matrix. However, close to the surface of the inner membrane bearing negative charge, there are few layers of structured water, which serve as a conductor for protons by the Grotthuss mechanism, and thus the local concentration of protons may be several orders higher than in the bulk volume of the matrix [21].

To explain the mechanisms of oxidative damage of cardiolipin and other mitochondrial phospholipids, we have suggested that low pH at the surfaces of the inner mitochondrial membrane, that is at the border between the lipid phase of the membrane and the water phases of the matrix and intermembrane compartments, is of paramount significance for the conversion of the relatively chemically inactive superoxide radical (O_2_^•^) to the highly active perhydroxyl radical (HO_2_^•^) [12, 17].

## 4. Properties of the Perhydroxyl Radical (HO_2_^•^)

**Figure 2:**
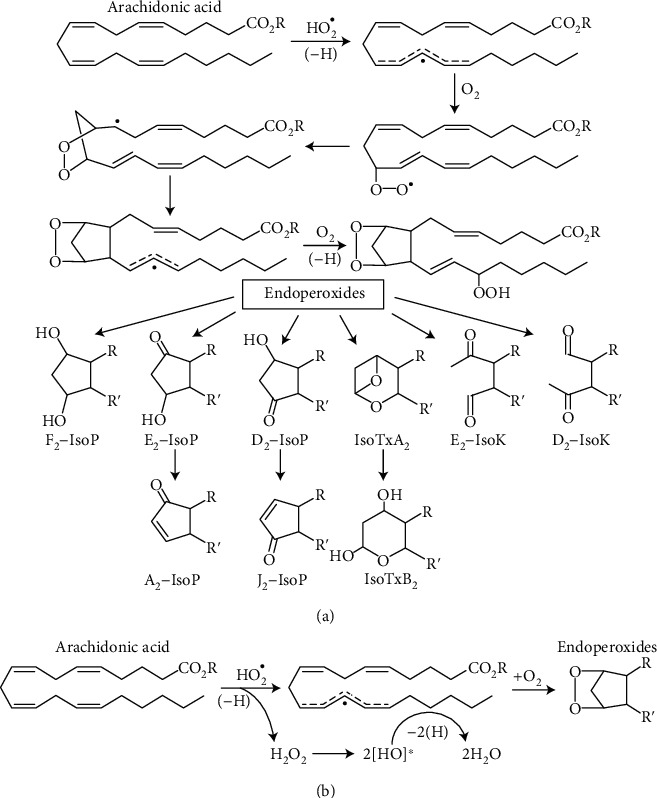
Autoxidation of arachidonic acid with rearrangements into different ring structures. (a) This part of Figure 2 (adapted from [81]) presents possible intermediate metabolites during autoxidation of arachidonic acid (AA) by some radical. HO_2_^•^ is the only candidate to initiate autoxidation of AA that is esterified to a phospholipid [17]. (b) The proposed sequence of transformation of the HO_2_^•^ and AA during IPLP [17]. Upon abstraction of the 1^st^ H atom from AA, HO_2_^•^ turns into H_2_O_2_, which in the hydrophobic environments undergoes homolytic decomposition into two molecules of ^•^OH radical, which instantly abstract additional two H atoms from AA, producing 2H_2_O and the remnant of AA with complete disarranged double bonds. The extremely fast abstraction of three H atoms from any two double bonds creates a highly unstable molecule of AA, which very rapidly and randomly reacts with two molecules of O_2_ and undergoes intramolecular rearrangements, which results in a large number of positional isomers and stereoisomers. The more PUFA has double bonds, the larger is the number of positional isomers and stereoisomers. Abbreviations: F_2_-IsoP, E_2_-IsoP, D_2_-IsoP are Isoprostanes with rings, correspondingly F_2_, E_2_, D_2_, or A_2_ and J_2_; IsoTxA_2_ and IsoTxB_2_ are isothromboxanes with rings A_2_ and B_2_, correspondingly, formed from Prostanglandin-H_2_ (PGH_2_); E_2_-IsoK and D_2_-IsoK are Isoketals with rings E_2_ and D_2_.

Superoxide radicals and hydrogen peroxide are the most common oxidants produced by mitochondria [47, 48]. Superoxide radical, when formed in the mitochondrial membrane, is rapidly removed from the lipid phase into the matrix or the intermembrane space because it is anion [49]. For some time, O_2_^•^ was considered as the main radical responsible for oxidative stress and aging, but soon, it was discovered that O_2_^•^ very poorly interacts with polyunsaturated fatty acids (PUFA) and amino acids [11, 18]. Therefore, the suspects became oxidants, which may originate from superoxide radicals, such as peroxynitrite (ONOO¯) and hydroxyl radical (^•^OH). Peroxynitrite, as well as peroxynitrate (O_2_NOO¯), react relatively slowly with most, but not all, biological molecules, making them rather selective oxidants. Both ONOO¯ and O_2_NOO¯ modify tyrosine in proteins, leaving a footprint detectable *in vivo* [50–52]. The most active oxidant, the ^•^OH, indeed is so active that it reacts within 1 to 5 molecular diameters of their site of formation [53]. According to Pryor [53], ^•^OH reacts with free linoleate with rate constants that are nearly diffusion-controlled. The lifetime of ^•^OH radicals have been estimated 10^−9^ sec. It reacts indiscriminately with organic molecules/groups and, therefore, cannot reach the inner layer of lipid membrane containing the unsaturated double bonds to initiate lipid peroxidation. All of the above mentioned radicals are hydrophilic. Perhydroxyl radical is a protonated form of the superoxide radical and has molecular formula HO_2_^•^, and it is hydrophobic and much more active chemically than O_2_^•^. Unlike the superoxide (O_2_^•^), HO_2_^•^ is a powerful oxidant [53]. The above considerations leave us with the only plausible candidate for initiation of the autoxidation of polyunsaturated fatty acids (PUFA) and CL, the perhydroxyl radical (HO_2_^•^) [12, 17] (Figure 2).

Perhydroxyl radical is always present in the cell due to reversible reaction ${{\text{O}}_{2}}^{\underset{¯}{•}}+{\text{H}}^{+}↔\text{H}{{\text{O}}_{2}}^{•}$ with pKa = 4.88 [54]. Because of the low pKa, it was widely accepted that at pH 7.2 in the cytoplasm, much less than 1% of [O_2_^•^] is present as HO_2_^•^ [55]. Perhaps for this reason, many researchers presumed that HO_2_^•^ has little or no role in initiation of lipid peroxidation [56]. However, several authors pointed out that pH values in the microvolumes in the vicinity of charged membranes may be several units lower than in the bulk volume of a cell [18, 21, 57]. This may occur around the negatively charged heads of cardiolipin, and other phospholipids, such as phosphatidylserine and phosphatidylinositol that may retain protons. Thus, not the bulk pH, but local pH at the interfaces of the IMM in matrix and cytosol is critical for formation of HO_2_^•^, and because it has no charge, HO_2_^•^ can easily go back into the lipid core of the membrane and cross it [58]. It should be also kept in mind that on both sides of the IMM, protons have much higher mobility by the Grotthuss mechanism in the few layers of structured water molecules close to the charged surfaces of the membrane, particularly around the rafts with antennae made of cardiolipin [21, 59, 60]. It is important that interaction of O_2_^•^ with H^+^ occurs at the interfaces of the matrix and cytoplasmic sides of the IMM where CL clusters hold together respirosomes and thus where superoxide radicals are produced [47]. For this reasons, CL and PEA may be the first phospholipids with unsaturated bonds affected by HO_2_^•^.

In comparison with other oxidants, HO_2_^•^ shows high specificity in reaction with PUFA, linoleic (C18:2), and linolenic (C18:3) acids [11]. Thus, when HO_2_^•^ encounters PUFA attached to a phospholipid, or two double bonds of linoleic acids (18:2) of CL, it reacts with them, with high probability and fast. This might explain why CL is one of the most sensitive phospholipids for oxidative damage [46, 61, 62]. Indeed, it has been shown that increased production of mitochondrial HO_2_^•^ in cardiovascular conditions is associated with an increased cardiolipin oxidation, which represent a specific biomarker of mitochondrial oxidative stress [63]. The outcome of HO_2_^•^ reaction with a different number of double bonds will be very distinct: IPLP, which requires PUFA with at least three double bonds present, produces racemic mixtures of hundreds of final products, whereas reaction with the most common fatty acid in CL, linoleic acids with two double bonds, yields “regular” lipid hydroperoxide [64].

## 5. The Isoprostane Pathway of Lipid Peroxidation

More than 50 years ago, it was observed that during autoxidation of linolenic acid or prolonged storage of the human blood plasma at -20°C, there were formed products similar to prostaglandins H and F_2_ [65]. Roberts and Morrow at the Vanderbilt University have shown the nonspecific auto-oxidative formation of prostaglandins *in vivo* [15, 16, 66–68] and discovered the nonenzymatic pathway of PUFA lipid peroxidation with racemic mixture of products possessing enormous variations in molecular positional isomerism and stereoisomerism in structure and biological activities.

A large number of products of this type of PUFA autoxidation possess high reactivities with lipids and proteins, and the resulting products may be considered as the most reliable and sensitive early markers of oxidative damages of lipids and proteins during aging and aging-associated pathologies [68–71]. The major features of the IPLP type of lipid peroxidation have been described in a number of publications [16, 66, 72]. The isoprostanes (IsoPs) that contain F-type prostane rings analogous to PGF_2_ were the first class of oxidation products discovered during IPLP in abundance *in vitro* and *in vivo* [71].

IsoPs and the cyclooxygenase-derived prostaglandins (PGs) have a number of distinctions in their origin and properties, which have been discussed in a number of publications [12, 66, 71, 73–76]. Here we briefly list the most important distinctions: (1) The side chains of PGs are almost always oriented *trans* to the prostane ring whereas the products of IPLP have mostly the side chains with *cis* orientation [66, 71]. (2) The IsoPs are formed *in situ* from PUFA, which are esterified to phospholipids, while PGs are generated exclusively from the free AA and DHA [74]. (3) The products of IPLP are the racemic mixture of products with a very large number of possible stereoisomers and positional isomers, whereas the products of the enzymatically produced prostaglandins have only one optical isomer each [66, 74].

Some of the products of PUFA autoxidation, such as *γ*-ketoaldehydes, are highly reactive molecules, which form adducts with primary amines of the lysine-containing proteins and phosphatidylethanolamine [69]. The most active among *γ*-ketoaldehydes formed from AA via the IPLP are isolevuglandins (IsoLGs). IsoLGs are so reactive, that were revealed only as adducts with proteins or ethanolamine of PEA. A number of IsoPs possess potent biological activity and thus can function as mediators of the oxidant injury, or convey abnormal cellular signaling [69].

**Figure 3:**
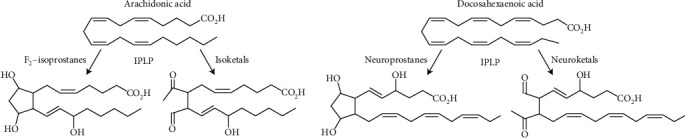
Examples of the parent and the corresponding product molecules resulting in the loss of two double bonds during nonenzymatic IPLP. During IPLP, the parent arachidonic and docosahexaenoic acids lose two unsaturated bonds. From AA may be produced various F2-isoprostanes and isoketals; from DHA, correspondingly, neuroprostanes and neuroketals. The images of the parent and one of the product molecules were adapted from [76].

In addition to arachidonic acid (AA), which is the most common among phospholipids, other PUFA such as eicosapentaenoic acid (EPA) and docosahexaenoic acid (DHA) have been found as substrates for the IPLP [70]. Because DHA is present in a larger quantity in neurons, the products of IPLP were correspondingly named neuroprostanes and neuroketals (Figure 3).

The differences between IPLP and the “classical” lipid peroxidation have been discussed in [17]. However, the mechanisms, which are responsible for such enormous diversity of stereoisomers and positional isomers of the final product of PUFA during the IPLP, remained unknown, as well as the radical responsible for initiation of IPLP.

Figure 2(a) illustrates one of the common presentations of PUFA autoxidation. Initially, the radical that initiates autoxidation of AA was denoted as ^•^OH [76], Morrow and Roberts usually showed no radical at all [66, 74, 75]. The problem with hydroxyl radical is that it cannot by itself appear in the middle of the hydrophobic core of the membrane. HO_2_^•^ is the only radical that can spontaneously come in contact with a PUFA, which is part of a phospholipid.

Recently, a hypothesis on the mechanism of initiation of IPLP by perhydroxyl radical (HO_2_^•^) was presented [12, 17]. If we accept that it is the perhydroxyl radical that abstracts the first H atom from AA, then the product of the reaction will be hydrogen peroxide (H_2_O_2_). It has been shown [77, 78] that in the hydrophobic milieu, H_2_O_2_ undergoes homolytic splitting with formation of two hydroxyl radicals: H_2_O_2_⟶2^•^OH, which instantly will abstract another two H atoms, more likely from the same PUFA. This will delocalize all electrons in the double bonds with unpredictable structure of intermediate forms. Therefore, the intermediate structures of AA shown in Figure 2(a) above “endoperoxides” have no sense because nobody knows what structures will be acquired by intermediate metabolites. Therefore, in Figure 2(b), we show only the sequence of chemical intermediates for the perhydroxyl radical and show only the starting molecule AA and the possible structures of the final products. The proposed mechanism of IPLP initiation was first presented in [12, 17].

Although the abstractions of the three H atoms are shown schematically as separate and consecutive events, see Figures 2(a) and 2(b) and [77], in reality, abstraction of all hydrogen atoms occurs extremely fast, as one chain reaction, before two O_2_ molecules join the remaining skeleton of AA. Rapid abstractions of three H atoms occur randomly at any double bond, this makes the molecule of PUFA highly unstable. For this reason, the O_2_ molecules bind randomly with formation of variants of regioisomers in accordance with the number of double bonds in the parent PUFA and surrounding conditions. The subsequent intramolecular rearrangements also occur randomly with the formation of different variations of the final product. Figure 3 shows a comparison of parent molecules of AA and DHA with the structures of the resulting possible products. For AA, these may be F2-isoprostanes and highly toxic isoketals, and for DHA, correspondingly, neuroprostanes and neuroketals (Figure 3). The products of IPLP have *cis* configuration relatively to the cyclopentane ring.

According to Antonenko et al. (2008) in experiments *in vitro*, cardiolipin is highly sensitive to peroxidation in the presence of ^•^OH [44]. The principal distinction between the *in vitro* conditions described for ^•^OH and HO_2_^•^ radicals [18, 79], and the interactions of HO_2_^•^ with AA, or ^•^OH with CL, is that *in vivo* reactions proceed in the completely hydrophobic environment. According to Gebicki and Bielski [18], in the water-ethanol mixture, the reactions proceed in consent with the reaction sequence of the “classical” lipid peroxidation (LP), and abstraction of the first hydrogen atom from linoleic acid results in formation of hydrogen peroxide: LH +  ^•^HO_2_⟶L^•^ + H_2_O_2_, with the final formation of the stable end product linoleic hydroperoxide: LOO^−^ + LH^•^⟶LOOH + L^•^ [18]. Abstraction of hydrogen atom from linoleic acid by ^•^OH results in the conversion of the ^•^OH radical to water: LH +  ^•^OH⟶L^•^ + H_2_O. Bielski et al. [11] have concluded that reaction of HO_2_^•^ radical with a double allylic H atom of a PUFA is proportional to the number of double allylic H atoms, which makes PUFAs highly specific targets with high affinity for perhydroxyl radicals [11, 18]. No selectivity was observed under similar conditions with hydroxyl radicals, which abstract H atoms randomly. According to Roberts and Morrows group [66, 74, 75], the reaction of HO_2_^•^ with linoleic, linolenic, and arachidonic acids in water-ethanol solution, the formation of a stable products, which were the corresponding hydroperoxide [11], was relatively slow. On the contrary, studies on IPLP suggest that in the fully hydrophobic environment, reactions of HO_2_^•^ with PUFA and linoleic acid are extremely fast, probably because of formation of hydroxyl radicals inside the hydrophobic milieu. For this reason, we can consider ^•^HО_2_ as a “carrier” of 2 ^•^OH inside the lipid core of the membrane.

**Figure 4:**
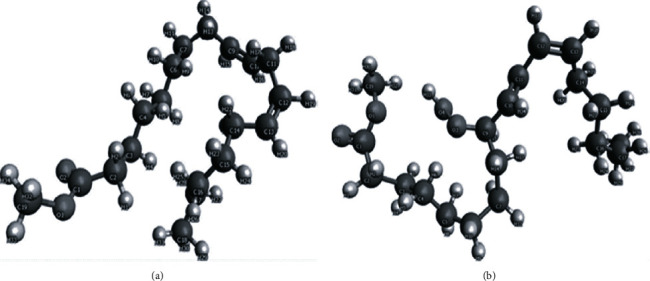
Conformational changes of linoleate molecule upon peroxidation. Molecular dynamic simulation of (a) methyl linoleate molecule conformation and (b) methyl linoleate hydroperoxide molecule. The figure was adapted from [68].

As a result, the selective peroxidation of CL during aging, which we presume is driven to a large degree by formation of HO_2_^•^ from the superoxide radical (O_2_^•^), abstraction of the first H atom from CL results in formation of hydrogen peroxide. In the hydrophobic milieu, H_2_O_2_ undergoes homolytic splitting into two molecules of ^•^OH, which instantly interact with another two H atoms of the same or adjacent linoleic acid followed by interaction of O_2_ with formation of the stable linoleic hydroperoxide, which is still attached to CL. Molecular dynamic simulation studies have shown that upon oxidation, the molecule of linoleic acid changes its conformation (Figure 4) [64], which may affect the ability of CL to maintain the superstructure of the mitochondrial enzyme complexes. Thus oxidatively damaged CL is a specific hallmark for the oxidative stress in mitochondria.

## 6. IPLP as the Mechanism of Aging

Numerous studies have demonstrated that IsoPs are the most early and reliable among available markers of lipid peroxidation *in vivo*, and recent studies provided valuable information about participation of IPLP in pathogenesis of numerous human diseases [15, 16, 68, 69]. According to our model of IPLP initiation by HO_2_^•^, the perhydroxyl radical upon encounter with a PUFA produces one of many variants of isoPG, iso-*γ*-ketoacids, or iso-levuglandins. When reacting with fatty acids with two unsaturated bonds, such as linoleic acids of cardiolipin, HO_2_^•^ produces corresponding hydroperoxides.

A large number of data proves that oxidative damages to cardiolipin [63, 80, 81] and phosphatidylethanolamine (PEA) [82, 83] may serve as markers for the mitochondrial autoxidation caused by aging. Above, we have presented our arguments that the major mechanism of oxidative damages of fatty acids esterified with phospholipids is associated with formation of HO_2_^•^ during “normal” production of superoxide radicals. The properties of perhydroxyl radical make it clear that regardless how small may be production of HO_2_^•^, it will cause some direct damages to mitochondria and other cellular membranes, or via the formation of adducts with PEA and lysine-containing proteins [82, 83], and peroxidation of cardiolipin [46, 63, 80, 81]. Even though the level of HO_2_^•^ production may be very low, it is, probably, the major mechanism of small, but persistent, accumulation of damages and regulatory signals caused by iso-prostaglandins, which, probably, often are wrong signals. Taking into consideration the crucial roles of CL and PEA in maintaining the structures of respiratory complexes and other polyenzymatic complexes, we can state that during aging the primary causes of mitochondrial dysfunctions are not limited by mutations of mtDNA, but more likely are caused by the functional and structural changes of phospholipids and proteins of mitochondria themselves.

## 7. The Importance of Fatty Acids Oxidation for Increased Rate of ROS Production

Mammalian mitochondria generate superoxide and hydrogen peroxide (ROS) from at least 11 different sites associated with substrates catabolism and the electron transport chain [48]. All mitochondrial sites of ROS production have very distinct properties [48, 84]. They can be divided into two groups: six sites operate at the redox potential of the NADH/NAD^+^ isopotential pool, about -280 mV, and five sites operate at the redox potential of the ubiquinol/ubiquinone (QH_2_/Q) isopotential pool, about +20 mV [48, 84].

The increased mitochondrial respiration can result in either increased production of ROS, if the accelerated respiration was caused by increased substrate supply, or in a decrease of ROS production, if the accelerated respiration was the result of increased utilization of ATP [48]. Heart, skeletal, muscles, and brain may work at very different workloads. In order to produce more ATP during high workloads, the mitochondria must receive the correspondingly increased supply of electrons into the respiratory chain. The commonly used substrates, often regarded as complex I substrates, such as glutamate and pyruvate, have relatively low rates of respiration because the NADH dehydrogenase of complex I is the rate limiting step [85]. The accelerated rates of respiration may be achieved by activation of glutamate and pyruvate transamination, or by the use of substrates mixtures, such as pyruvate + glutamate + malate (for the brain). Under these conditions, activation of transaminase reactions in mitochondria produce, in addition to NADH, 2-oxoglutarate, which is then converted to succinate in the TCA cycle [85–87]. Brand has stressed the importance of fatty acids oxidation for the bioenergetics of the skeletal muscle and heart mitochondria [48, 89]. During *β*-oxidation of fatty acids by the multienzyme complexes, the electron-transferring flavoprotein-ETF:Q oxidoreductase system reduces the membrane's pool of ubiquinone. In the presence of palmitoyl-carnitine and other substrates (glutamate, pyruvate or succinate), there is no inhibition of SDH (complex II) [88], and thus electrons enter the respiratory chain both through the NADH/NAD^+^ and QH_2_/Q pathways allowing at high workloads fast production of ATP [48, 89, 90]. If consumption of ATP is limited, the excessive electrons activate at high membrane potential the reverse electron transport, which results in increased production of superoxide and H_2_O_2_ [48, 89]. We have suggested that in people with metabolic syndrome, which is characterized by increased utilization of fatty acids for production of energy, aging of the heart and brain may be accelerated [88, 90]. Indeed, recent studies support the cross-talk between the oxidative stress and metabolic conditions in mitochondria [91].

## 8. Oxidative Stress and the Mitochondrial Membrane Integrity

Mitochondria, particularly the inner membrane, contain very large amount of proteins, many of them in hundreds and thousands copies. Between proteins are located phospholipids, which comprise only around 20–25% of the total mass. Cardiolipin is located almost exclusively in the inner membrane of mitochondria (IMM). However, only phosphatidylcholine, together with few other phospholipids, form a biological membrane. Interactions of phosphatidylethanolamine (PEA) and cardiolipin (CL) with proteins allow integration of large proteins and multiprotein complexes into numerous curves of the inner membrane. PEA has a conical form because at C2, this phospholipid has a polyunsaturated fatty acid, usually arachidonic acid (С20:4, *ω*-6) or docosahexaenoic acid (С22:6, *ω*3), which have a curved shape. During oxidative stress, due to the high affinity of HO_2_^•^ to polyunsaturated fatty acids, PEA also undergoes peroxidation as well as the linoleic acid of cardiolipins. These two phospholipids are located close to each other because they share the function of maintaining the structural integrity of the IMM and multienzyme complexes. Therefore, oxidative damages to CL and PEA result in the malfunctions of the lipid-protein interactions and dysfunction of proteins. Accumulation of oxidized PEA and CL and their depletion are mitochondrial hallmarks of aging [80, 92, 93]. Recently, it has been shown that during aging and diabetes, the fatty acid composition of CL may undergo remodeling when C18:2 fatty acids can be replaced by arachidonic acid (С20:4, *ω*-6) or docosahexaenoic acid (С22:6, *ω*3) [94, 96], which are substrates for IPLP [13, 14]. Because CL is directly involved in formation of HO_2_^•^, it may explain why CL and PEA are particularly sensitive to oxidative damages [62, 92, 93].

It is conceivable that oxidative damages of even small amounts of CL and PEA may cause significant dysfunctions in energy production by mitochondria because both phospholipids are responsible for the superstructural organization of proteins involved in oxidative phosphorylation [96, 97]. In addition, CL is tightly bound with the transmembrane carriers of respiratory substrates, cytochrome *c*, and ANT. In the absence of CL, the electron transport between the respiratory complexes becomes disrupted, the membrane potential drops significantly, and the synthesis of ATP becomes inhibited [98, 99].

In comparison with CL, the effects of PEA on mitochondrial functions are less studied. However, it was established that in the absence of PEA, the transport and assembly of proteins are also abnormal, only in this case, with the presence of only CL, the proteins tend to form unusually large superstructures, which result in the loss of membrane potential and ATP synthesis [98, 99]. Normal structural organization and functioning of respiratory chain and ATP-synthase requires coordinated interactions between CL and PEA.

## 9. Aging, Oxidative Stress, and Metabolic Syndrome

Oxidative stress is regarded as the main cause of aging, mitochondrial dysfunctions, and thus is one of the main pathogenic mechanisms of many diseases [100–105]. This is particularly true for those diseases, which are associated with the metabolic syndrome that develops at the certain stage of aging in humans [100–102]. It is clear, however, that the metabolic syndrome is not the result of only accumulated errors and dysfunctions in the course of life for whatever reason. From the point of view of the normal ontogenesis of an individual after birth, development of the metabolic syndrome represents a normal metabolic transition from the reproductive to post productive state of an individual.

**Figure 5:**
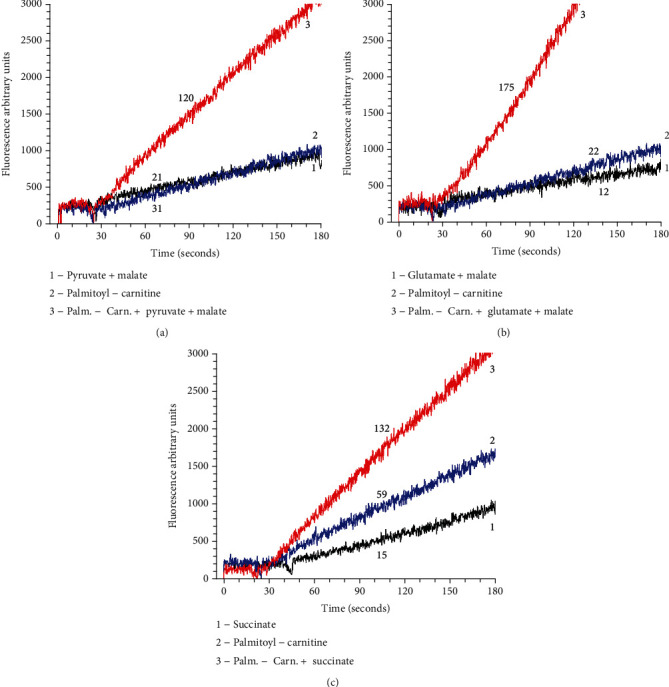
Production of superoxide radicals by rat heart mitochondria-oxidizing palmitoyl-carnitine. Designations: 1. supporting substrate only; 2. palmitoyl-carnitine only, and 3. palmitoyl − carnitine + supporting substrate. Substrates: Figure 5(a) (pyruvate 2.5 mM + malate 2 mM), Figure 5(b) (glutamate 5 mM + malate 2 mM), and Figure 5(c). (succinate 5 mM). Experimental conditions are described in [93]. The incubation medium contained Amplex Red 2 *μ*M, horse radish peroxidase 2 units, substrates as indicated above, and volume 1 ml. The reaction was initiated by addition of 50 *μ*g of mitochondria. Initial rates were measured for 3 minutes. Numbers at the traces are the rates of H_2_O_2_ production in picomol H_2_O_2_/min/mg protein RHM. The rates were corrected for the time control rate with RHM incubated without added substrates. The figure was taken from [95].

Recently, we have shown that the full-scale oxidation of fatty acids by synaptic brain and heart mitochondria at all metabolic states occurs synergistically in the presence of other mitochondrial substrates, such as pyruvate, glutamate, or succinate [88]. We have also shown that oxidation of fatty acids may cause a several-fold increase in production of ROS [88, 90].  Figure 5 illustrates how simultaneous oxidation of palmitoyl-carnitine and other substrates affects production of ROS.

One of the important features of the metabolic syndrome is a dramatic increase in utilization of fatty acids for production of energy by mitochondria, particularly in women after menopause [106, 107]. From Figure 5, we can deduce that in aged individuals with metabolic syndrome, increased utilization of fatty acids may cause increased oxidative stress and thus accelerate the rate of aging.

## 10. The Rate of Aging is Proportional to the Rate of Superoxide Radical Production

Formation of HO_2_^•^ is proportional to the level of O_2_^•^ present at the interfaces of the inner membrane with the matrix and intermembrane spaces, where concentration of O_2_^•^ at any moment is determined by the rates of its production and elimination [48]. Taking into consideration that HO_2_^•^ is extremely reactive and dangerous, it is understandable that removal of superoxide radicals is very important for protecting cells from the deleterious effects of its protonated form as ^•^HО_2_. The activities of SOD2 and SOD1 are of most importance for the heart and the central nerve system, where the alternative antioxidant systems are relatively weak, whereas the contents of AA and DHA are the highest [33]. Because HO_2_^•^ interacts with PUFA and CL inside the membranes, any type of antioxidants, including superoxide dismutases, will have no effect on the aging caused by HO_2_^•^ but will have effects on aging processes caused by other radicals. This means that the rate of HO_2_^•^ formation is strictly dependent on the rate of superoxide radical formation. As we have discussed above, at old age, when fatty acids become the predominant substrates for energy provision, the rate of aging may also increase. Thus, to delay aging, first of all, it is necessary to delay production of superoxide radical, and secondly, to diminish conversion of superoxide into ^•^HО_2_. Local cellular conditions also affect the amount of O_2_^•^ converted to HO_2_^•^, for example, accumulation of lactic acid during high physical loads or mild hypoxia may increase oxidative damages to skeletal muscle cells or cardiomyocytes due to the acidification-induced higher levels of HO_2_^•^ production [17]. From this point of view, of particular interest for our discussions are recent publications of the Skulachev's school of researchers on the effects of the mitochondria-targeted antioxidants with plastoquinone as an electron acceptor [46, 98–111].

Summarizing the data of several publications of the Skulachev's research team, it has been concluded [98] that the plastoquinone-based mitochondria-targeted antioxidants operate in two quite different ways: (i) by preventing peroxidation of cardiolipin [46, 99] and (ii) by mild uncoupling of mitochondria resulting from fatty acid cycling that inhibits the formation of reactive oxygen species in mitochondria [111].

Shabalina et al. (2017) used the mtDNA mutator mice, which exhibit marked features of premature aging. The authors have suggested that accelerated aging was caused by the increased mitochondrial ROS that interact with polyunsaturated fatty acids in cardiolipin, releasing malondialdehyde and 4-hydroxynonenal that form protein adducts and thus diminish mitochondrial functions [109]. Treatment of animals with accelerated aging with SkQ1 counteracted these changes as it scavenges mitochondrial ROS. As the results, the normal mitochondrial ultrastructure was preserved in liver and heart; the phosphorylation capacity of skeletal muscle mitochondria as well as the thermogenic capacity of brown adipose tissue also improved. The SkQ1-treated mice lived significantly longer (335 versus 290 days) [109].

## 11. Conclusions

In this review, we examined the unique structure of cardiolipin and how this structure is reflected in several unique functions of this phospholipid. Cardiolipin (CL), together with phosphatidylethanolamine (PEA), maintain the superstructural organization of the enzymes that perform the most important function of mitochondria–oxidative phosphorylation. Both CL and PEA are not capable to build up a flat biological membrane, but they accommodate the functional complexes of proteins into the sharp curves of the inner mitochondrial membrane. At some regions of the IMM, cardiolipin creates regions with a strong negative charge, where local pH may be several units lower than in the bulk phase of a compartment. We suggest that at these regions, the probability of protonation of the superoxide radical is relatively high. The resulting molecule of perhydroxyl radical is hydrophobic and specifically reacts with the double bonds of PUFA and linoleic acid of cardiolipin, producing correspondingly racemic mixture of isoprostanes and isoketals from PUFA and linoleate hydroperoxide. This explains why CL and PEA are the most common markers of the specific oxidative damages in mitochondria. Damages to CL and PEA cause gradual disarrangement of the structural organization of the functional complexes of oxidative phosphorylation as well as other enzymes of the mitochondria. For this reasons, we consider that oxidative damages to CL and PEA are the primary cause of aging and the age-associated diseases. Therefore, developments of the mitochondria-targeted antioxidant drugs, similar to SkQ, are very promising in finding and making a reality the existence of the mythical “Elixir of Youth.”

## Abbreviations

AA: Arachidonic acid (C20:4 *ω*6)
ANT: Adenine nucleotide translocase
CL: Cardiolipin
COX: Cyclooxygenase
DHA: Docosahexaenoic acid (C22:6 *ω*3)
HO_2_^•^: Perhydroxyl radical
IMM: Inner mitochondrial membrane
IsoPs: Isoprostanes
IPLP: Isoprostane pathway of lipid peroxidation
LOX: Lipoxygenases
LP: Lipid peroxidation
MetS: Metabolic syndrome
mtDNA: Mitochondrial deoxyribonucleic acid
NO: Nitric oxide radical
O_2_^•^: Superoxide radical
OH: Hydroxyl radical
OMM: Outer mitochondrial membrane
ONOO¯: Peroxynitrite
O_2_NOO¯: Peroxynitrate
OXPHOS: Oxidative phosphorylation
PEA: Phosphatidylethanolamine
PGF_2_: Isoprostanes containing F-type prostane rings
PGs: Prostaglandins
PUFA: Polyunsaturated fatty acids
RET: Reverse electron transport
ROS: Reactive oxygen species
SOD: Superoxide dismutase
SOD1: Cytoplasmic isoform of Cu,Zn-SOD
SOD2: Mitochondrial isoform of Mn-SOD.
